# Human rabies post exposure prophylaxis at the Pasteur Institute of Dakar, Senegal: trends and risk factors

**DOI:** 10.1186/s12879-019-3928-0

**Published:** 2019-04-11

**Authors:** Mamadou Korka Diallo, Alpha Oumar Diallo, Anta Dicko, Vincent Richard, Emmanuelle Espié

**Affiliations:** 1 Pasteur Institute of Dakar, Centre for human rabies post exposition prophylaxis, Dakar, Senegal; 2 Pasteur Institute of Dakar, Epidemiology unit, Dakar, Senegal; 3 Department of studies and research in statistics and development, University Gaston Berger of Saint Louis, Saint Louis, Senegal

**Keywords:** Rabies, Post exposure prophylaxis, Dog bites, Risk factors, Knowledge, Sub-saharan Africa

## Abstract

**Background:**

Rabies remains a major public health problem in developing countries. Most fatal rabies cases, especially in children, result from dog bites and occur in low-income countries, such as those in Sub-Saharan Africa. Rabies can be controlled through mass dog vaccination and human deaths prevented through timely and appropriate post-exposure prophylaxis (PEP). As access to appropriate PEP remains a serious challenge for bite-victims, the aim of this study was to understand the use of PEP, to evaluate the knowledge, attitudes and practices with respect to rabies and to identify risk factors related to non-compliance with PEP to define recommendations for improving PEP in Senegal.

**Methods:**

This study included patients with suspicion of rabies exposure who sought PEP at the Pasteur Institute of Dakar from April 2013 to March 2014. Patients with rabies clinical symptoms, those who had already started PEP and those with exposure outside Senegal or for more than 3 months were excluded. Data on risk factors and propensity to seek and complete PEP were collected using questionnaires and phone interviews. The association between acceptability and compliance with PEP and other independent variables were evaluated using multivariate regression analysis.

**Results:**

Among the 905 patients enrolled into the study, 67% were male (sex ratio M/F, 2) and 46%, children under 15 years of age. Exposures by animal bites represented 87%, whereas the remainder were due to scratches or contact; 76% were classified as WHO category III and 88% were due to dogs. Among these patients, 7% refused to start PEP and 54.5% completed the full schedule. Main factors reported by non-compliant patients were vaccine costs and affordability, and knowledge on status of biting animal.

**Conclusion:**

This study shows that despite the awareness about rabies dangers and prevention, only half of the patients completed the full PEP schedule. The following recommendations, such as free of charge prophylaxis or intradermal regimens as an alternative to intramuscular regimens, should be considered to increase the adherence to PEP at the Pasteur Institute of Dakar and in Senegal.

## Background

Rabies remains a major public health issue in the world, with 59,000 human deaths per year, mainly in low and middle income countries of Asia and Africa [1]. In Western and Central African countries, reporting of rabies is not mandatory, so few epidemiological data are available [2]. This has resulted in lack of prevention and control measures possibly due to under reporting, insufficient follow- up on victims of animal bites, lack of data on the public health impact of the disease, and absence of programs for effective vaccination of dogs.

Rabies is acute, progressive and is almost 100% fatal once clinical symptoms develop. However, the disease is preventable with timely implementation of vaccination after exposure to a suspected rabid animal (named Post-Exposure Prophylaxis, PEP). PEP equals more than 20 million treatments per year and has been an effective control measure to rabies for more than 100 years [3]. The WHO-recommended PEP consists of an immediate bite wound washing or flushing, administration of rabies vaccine and, when category III exposure is recognized additional injection of rabies immunoglobulin (RIG) [4, 5]. PEP should be started as soon as possible after a recognized exposure [6]. Several PEP regimens (using intramuscular or intradermal administration) are currently approved for individuals not previously vaccinated against rabies [5].

**Fig. 1:**
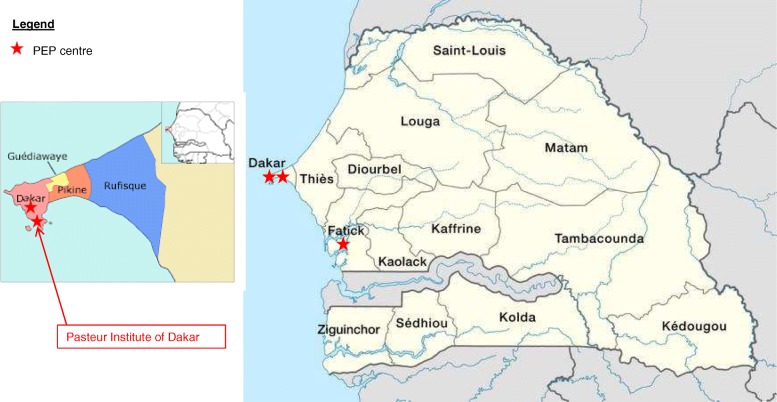
Map of Senegal with the location of the three rabies vaccination centres (named “PEP centre”). NordNordWest [under the Creative Commons Attribution-Share Alike 3.0 Unported], from Wikimedia Commons (https://commons.wikimedia.org/wiki/File:Senegal,_administrative_divisions_-_en_-_monochrome.svg)

In Senegal where rabies is endemic, human rabies is a notifiable disease. Between 1995 and 2016, 79 human cases were reported by the Fann University Hospital of Dakar [7]. Although the annual reported number of human rabies cases is very low across the country [7–9], the number of individuals seeking PEP after suspected exposure to the virus has increased in the past years. Since 1963 in Senegal, the Pasteur Institute of Dakar is one of the major public rabies vaccination centres. Despite the recent implementation of PEP in two other hospitals (the Fann University Hospital of Dakar in 2010 and the regional hospital of Fatick in July 2013), the Pasteur Institute of Dakar remains the most reliable data source to describe the use and administration of rabies PEP in Senegal (Fig. 1). Thus at the Pasteur Institute of Dakar, from 2008 to 2012, between 850 and 1000 patients have received PEP annually, but very few data on PEP use are publicly available. Moreover, the discrepancies observed between the expected number of vaccine doses that should have been delivered if all patients who have been advised to start PEP were compliant, and the observed number of administered vaccine doses suggested that the implementation of PEP at the Pasteur Institute of Dakar was not optimal.

The aim of this study was to understand the use of human rabies PEP, to evaluate the knowledge, attitudes and practices of patients with respect to rabies, and to identify risk factors related to a partial PEP schedule, in order to propose recommendations for improving the PEP delivered to patients at the Pasteur Institute of Dakar, in Senegal.

## Methods

### Study design and study population

A prospective cohort study was carried out from April 1, 2013 to March 31, 2014, at the Pasteur Institute of Dakar. All patients reporting contact with suspected rabid animals and who had sought PEP at the Pasteur Institute were screened for eligibility in the study. Patients with rabies clinical signs, those who had already started PEP in another hospital and those who had been bitten more than 3 months ago or bitten outside of Senegal, were not considered eligible for this study.

When a patient was first admitted at the Pasteur Institute of Dakar, a clinical examination was performed by a physician who decided whether PEP was needed, based on WHO recommendations and the exposure context. If PEP was recommended, the physician informed the patient about the study objectives and study procedures (including potential phone calls). If the patient agreed to participate and had a mobile phone to be reachable during the study period, the physician obtained his/her consent. Children without legal representative or adults who refused to participate were excluded. For patients who signed the informed consent form, the physician completed the study questionnaire, explained the purpose of PEP and procedures and started the prophylaxis.

At the Pasteur Institute of Dakar, PEP protocol for unvaccinated individuals (Zagreb regimen) consists of injection of four intramuscular doses of a purified vero cell rabies vaccine (Verorab Sanofi Pasteur, France) at D0 (2 doses), D7 (one dose) and D21 (one dose) [4]. In case of severe wound, equine RIG (Favirab Sanofi Pasteur, France) are administered at the initiation of PEP, in addition of the two first doses of the vaccine.

At the initial visit (D0), a structured questionnaire was used to collect data including socio-demographic characteristics (education level, pets’ ownership, household size), description of the wound(s) (number, size, location), exposure (date and place, animal involved, WHO categories I, II, III [4]), treatment (local treatment of injuries, antibiotics administration, previous rabies vaccination), knowledge of rabies and attitudes in respect to animal bite.

At D7 (2nd visit) and D21 (3rd visit), follow-up questionnaires were completed by a nurse to collect additional data on potential occurrence of adverse effects after vaccination and current status of the suspected rabid animal. When patients didn’t attend the follow-up visits at the Pasteur Institute of Dakar, a phone interview was conducted within the 7 days after the planned visit in order to identify the reasons of the absence at the planned follow-up visit. For patients who came back to the Pasteur Institute of Dakar to continue PEP after the follow-up call, data were recorded, but not included in the statistical analysis.

### Statistical analysis

The compliance of PEP was defined as follows: “No PEP” if the patient didn’t start the prophylaxis, “Partial PEP” if the patient received two or three doses and “Complete PEP” if the patient received the full 4-dose schedule.

Proportions of PEP recipients by gender, age groups, season, socio-demographic factors, type of exposure (animal bite versus non-bite) and knowledge of rabies were estimated. Among patients who started PEP, differences between partial and complete PEP were assessed with Fisher’s test or Mann-Whitney test of significance. A multivariate logistic regression model was developed to identify possible risk factors for partial PEP, using a forward stepwise selection approach. Odds ratios (OR) with Wald 95% confidence intervals were estimated. Variables with *p*-values < 0.05 (based on the likelihood-ratio chi-squared test) in the multivariable model were considered to be significantly associated with partial PEP.

The main analysis was run on all subjects enrolled in the study, and a sensitive analysis was performed on subjects not related to a cluster, to investigate potential confounders.

Analyses were performed using STATA software version 10 (StataCorp).

### Ethical approval

The study protocol and the informed consent form were approved by the National Ethical Committee for Research of Senegal. The study was conducted in compliance with principles set out by the Declaration of Helsinki, and the regulatory requirements of the Senegalese government on Health Research Ethic. For children less than 16 years of age, individual written informed consent was obtained from all children’s parents or legal representatives, in the presence of an independent witness for illiterate parents/legal representative.

## Results

**Fig. 2:**
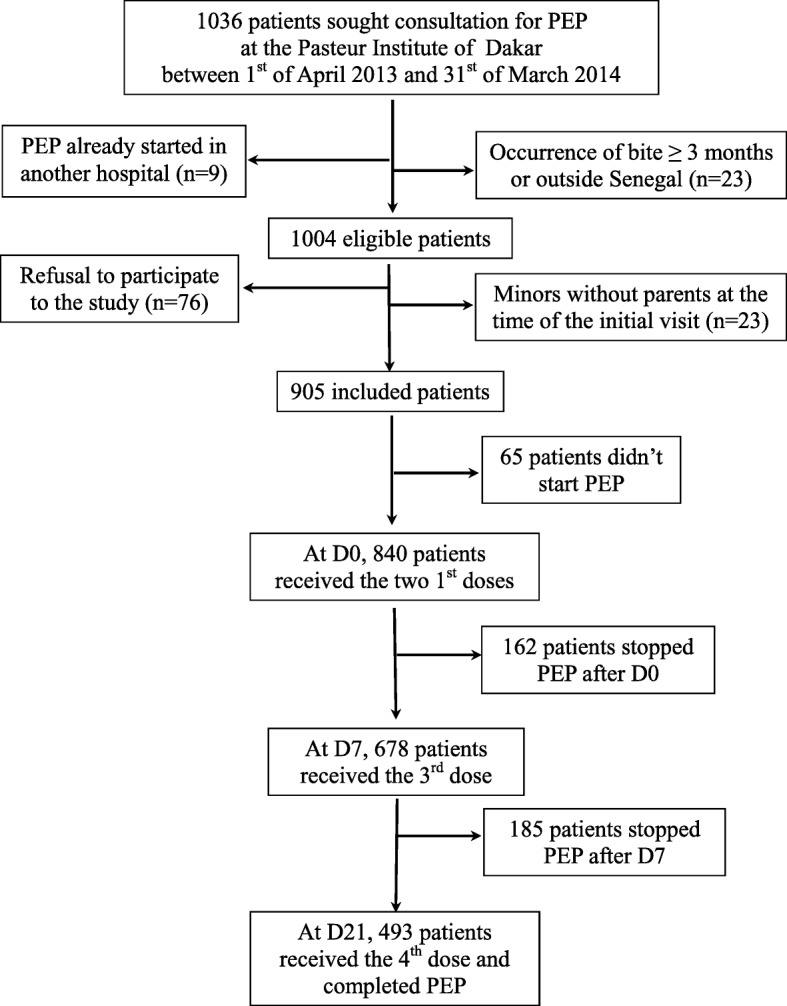
Flow chart of the patients who sought a consultation at the Pasteur Institute of Dakar for suspicion of rabies exposure, April 2013–March 2014

From April 1, 2013 to March 31, 2014, 1036 patients sought a consultation at the Pasteur Institute of Dakar for suspicion of rabies exposure. Among the 1004 eligible patients who were advised to get PEP, 905 (90.1%) were included in this study (Fig. 2). Among these patients, 66.8% were male (sex ratio M/F = 2.02), and 46.2% were children under 15 years of age. The mean age was 23.5 years [range: 14 months-80 years]. Eight hundred and forty-two individuals (93.0%) were exposed in Dakar and neighbourhood (Pikine, Rufisque and Guediawaye) and 93.7% were Senegalese (Table 1).

**Table 1:** Characteristics of the 905 patients and family households, according to PEP compliance

	Total cohort *N* = 905	No PEP *N* = 65	Partial PEP *N* = 347	Complete PEP *N* = 493
	n (%)	n (%)	n (%)	n (%)
Patient’s characteristics				
Male	605 (66.8)	50 (76.9)	240 (69.2)	315 (63.9)
Age, years (median, [range])	17 [1.2–80]	16 [2–61.5]	17 [1.5–80]	18 [1.2–78]
≤ 15 years of age	418 (46.2)	30 (46.2)	165 (47.6)	223 (45.2)
Senegalese nationality	848 (93.7)	63 (96.9)	328 (94.5)	457 (92.7)
Patient’s household characteristics				
High education level^a^ of the patient or family head	517 (57.4)	32 (49.2)	177 (51.0)	308 (63.0)
Patient or family head without job	160 (17.8)	12 (18.5)	52 (15.1)	96 (19.6)
Dogs ownership	225 (24.9)	10 (15.4)	88 (25.4)	127 (25.8)

*N* total number of individuals, *n* number of individuals in the specified category, *%* 100*n/N

^a^: The education level was considered as high if the patient or the family head graduated from the secondary school or studied at an Arabic school for more than 5 years or studied at the university at least one year

The most bitten body sites were legs (39%) and hands (21%). Eighty percent of patients reported more than two lesions (up to 12). Animal bites represented 87.2% of all exposures and 75.7% were WHO category III (Table 2). After exposure and before coming to the Pasteur Institute of Dakar, 496 patients went to a primary health care centre (e.g. clinic, health care centre, physician office), and 77% came the same day of the incident to receive preliminary treatment by a physician, a nurse or at a pharmacy.

**Table 2:** Post-exposure prophylaxis according to the site and time of exposures and characteristics of the suspected animal

	Total cohort N=905	No PEP N=65	Partial PEP N=347	Complete PEP N=493
	n (%)	n (%)	n (%)	n (%)
Type of exposure				
Simple contact/licking	37 (4.1)	1 (1.6)	4 (1.2)	32 (6.6)
Scratches only	78 (8.7)	6 (9.2)	25 (7.2)	47 (9.6)
Bite (with or without scratches)	785 (87.2)	58 (89.2)	318 (91.6)	409 (83.8)
Body parts bitten or scratched				
Legs-feet	446 (52.0)	36 (57.1)	172 (50.3)	238 (52.6)
Hands-arms	282 (32.9)	20 (31.8)	113 (33.0)	149 (33.0)
Head (including face)	39 (4.5)	0 (0)	23 (6.7)	16 (3.5)
Other (including trunk)	90 (10.5)	7 (11.1)	34 (9.9)	49 (10.8)
Season of exposure				
Rainy season (June-Oct)	415 (45.9)	36 (55.4)	166 (47.8)	213 (43.2)
Dry season (Nov-May)	490 (54.1)	29 (44.6)	181 (52.2)	280 (56.8)
Number of wounds and/or bite				
From 1 to 5	783 (92.6)	58 (92.1)	306 (91.6)	419 (93.3)
More than 5	63 (7.4)	5 (7.9)	28 (8.4)	30 (6.7)
Suspected rabid animal				
Dog	794 (87.7)	59 (90.8)	309 (89.0)	426 (86.4)
Cat	67 (7.4)	2 (3.1)	21 (6.0)	44 (8.9)
Other (monkey, horse, rat)	44 (4.9)	4 (6.1)	17 (5.0)	23 (4.7)
WHO category of exposure				
I	11 (1.2)	1 (1.6)	2 (0.6)	8 (1.6)
II	206 (23.1)	16 (25.0)	73 (21.3)	117 (24.0)
III	677 (75.7)	47 (73.4)	267 (78.1)	363 (74.4)
Delay between the exposure and the start of PEP ≥ 1 day	167 (18.5)	65 (100)	59 (17.0)	43 (8.7)

*N* total number of individuals, *n* number of individuals in the specified category; %: 100*n/N

Exposure occurred at home for 41% of exposures. In 77% of the exposures, only one individual was bitten. However, in January 2014, a cluster of 33 individuals, with a confirmed rabies exposure, was reported involving both children and adults living in a compound house of a non-profit organization in charge of the education of abandoned children.

Most suspected rabies exposures were due to dogs (88%), particularly domestic animals or pets (65%) (Table 2); 4.3% (37/861) of them were up-to-date vaccinated against rabies.

In rare situations involving in total 51 patients (including the cluster of 33 individuals), the seven suspected animals were slaughtered or euthanized and their head were sampled by the Government veterinary services for laboratory confirmation. All animal samples were confirmed positive for rabies.

### Post-exposure prophylaxis implementation

Among the 905 included patients, 467 patients (51.7%) went to the Pasteur Institute of Dakar to seek a consultation within the 24 h after the bite. Out of the 905 patients who were advised to get PEP, 840 patients (93%) received the two first doses and 66 received also immunoglobulins at D0. Sixty-five (7%) patients refused to start PEP and did not receive any vaccine mostly because of financial reasons. However, among them two patients decided to start PEP later, 17 and 30 days after the first visit at the Pasteur Institute of Dakar.

Out of the patients receiving PEP (Fig. 2), 162 (18%) patients received two doses only at D0, 185 (20.5%) three doses at D0 and D7 and 493 (54.5%) completed the full 4-dose schedule. Among the 51 patients who were exposed to a confirmed rabid animal, 94.1% completed the full 4-dose schedule, one patient received 3 doses and two, only the two first doses. Most of the dose administrations were performed in time: 87.0% at D7 [range, 6–14 days] and 77.7% at D21 [range, 14–31 days]. After the follow-up calls, 40 (11.5%) patients came back for the follow-up visit in order to continue PEP, with a median delay of 18 days [range, 14–31 days] after D0, and of 22 days [range, 19–52 days] after D7. No death was reported during the study period.

The two main reasons reported by the patients or family for not being compliant to PEP regimens at D7 and D21 were: inability to afford PEP and suspected animal still alive more than two weeks after exposure (Table 3). Victims often quoted several reasons: 36.4% reported two reasons and 20.5% more than 2 (up to 4). Among the 302 non-compliant patients, 62 (20.5%) reported that they didn’t come for the last dose because the suspected animal was still alive at the date of the planned visit (D21) and the medical staff of the Pasteur Institute mentioned at D7 that the PEP could be discontinued. However, no formal recommendation was clearly provided to the patients. Thirty-four patients or family (11.2%) reported that PEP was too expensive and they were not ill at the time of the planned visit.

**Table 3:** Reasons reported by the 302 non-compliant patients or family who answered to the follow-up calls

Reason (by order of frequency)^a^	Number	%
Costs of the PEP were too expensive	131	43.4
Animal was still alive at the time of the follow-up call	93	30.8
Patient did not “feel ill” at the time of the planned visit	66	21.8
Medical staff of the Pasteur Institute of Dakar told during the previous visit (D7) that it was not necessary to continue the PEP if the animal was still alive at the time of the planned visit	62	20.5
Patient or family was not available to come to the Pasteur Institute of Dakar at the date of the planned visit (e.g. hospitalisation of the patient’s parent, or out of the region/country)	56	18.5
Patient or the patient’s family did not understand that the visits of D7 and D21 were mandatory to ensure the efficacy of PEP	22	7.2
Patient had an adverse event after the vaccine dose injection	5	1.6

^a^Patients or family reported several reasons for not being compliant to PEP

Adverse events were reported after the first two doses by 6% of the patients (42/678) (including 5 patients who also received equine RIG at D0), and after the third dose, by 3% (16/493). Most of them were minor: headache (46.5%), fever (31%) and pain at the injection site (22%), and mostly (74%) occurred on the same day of the vaccine injection (up to 7 days).

### Risk factors for partial PEP course

The sensitive univariate analysis identified the following risk factors for a partial PEP, when sporadic exposure: low level of education of the family head, normal behaviour of the suspected rabid animal at the time of the exposure, no wound-cleansing with antiseptic after exposure, and no immunoglobulins administration at D0 (Table 4).

**Table 4:** Risk factors for partial PEP vs. complete PEP (sensitive univariate analysis, *p*-value < 0.1)

	Categories	OR [95%CI]	p-value
Level of education of the family head	High	Ref.	-
	Low	1.49 [1.12-1.98]	0.006
Wound-cleansing with antiseptic after exposure	Yes	Ref.	-
	No	1.43 [1.01-2.03]	0.042
Behaviour of the suspected rabid animal at the time of exposure	Unusual	Ref.	-
	Normal	1.45 [0.97-2.17]	0.06
RIG administration at D0	Yes	Ref.	-
	No	3.16 [1.65-6.05]	< 0.0001

In multivariate analysis, after adjusting for covariates (including the fact to be part of a confirmed rabies cluster), low level of education (AOR, 1.59; 95%CI [1.20–2.11]) and absence of RIG administration at D0 (AOR, 3.29; 95%CI [1.72–6.27]) were significantly associated with an increased risk for non-compliant PEP schedule.

### Knowledge of rabies and health practices

The majority of the patients or family reported that they were aware that rabies is transmitted by bite (93%), that dogs are the main reservoir of rabies (99%) and that rabies is fatal (90%). Most of the subjects (99%) would seek treatment from a doctor or a hospital after being bitten by a dog, but only 51.5% of the respondents were aware that the Pasteur Institute of Dakar is one of the main centres where the PEP to prevent rabies is provided.

## Discussion

This study confirms that the epidemiology of bite and treatment for suspected rabies exposures in Senegal is similar to what is observed in other African countries: children and young men are the most exposed [3], and dogs are the main reservoir [10–12]. Most of the animals involved were domesticated with identified owners, but only 4.3% of them were up-to date with rabies vaccination. The low vaccination coverage could be the result of a lack of regulations enforcement and controls for pet ownership in Senegal.

Over 93% of exposures were reported in the urban area of Dakar and the three surrounding cities that are highly populated areas. This finding could be a result of rapid urbanisation with rapid immigration of stray dogs to urban areas to find food and an increased contact interaction between human and dogs [13, 14]. However, this pattern would better reflect the catchment’s area of the Pasteur Institute of Dakar, instead of the true geographic distribution of rabies exposure in Senegal.

This study shows that the compliance rate for the full PEP schedule, as recommended by WHO, is quite low (55%). Regression modelling showed that the two risk factors associated with a non-compliant PEP were the low level of education of the patient or family and the absence of RIG administration at D0. These findings are coherent with studies suggesting that education of parents plays a significant role with regards to higher child immunization rates [15, 16]. Moreover, people with higher education tend to know more about rabies [17, 18], while illiterate individuals tend to know less about rabies [19]. This might explain the low level of concern about the fatal risk and the importance of the PEP compliance. Regarding the absence of RIG administration as a potential risk factor for non-compliance, it might be explained by the fact that the administration of RIG is highly recommended in case of confirmed rabid exposure and severe wounds. In this specific situation, a more detailed counselling regarding PEP and its compliance might be performed compared to situations where RIG was not recommended.

Furthermore, the first reason highlighted by the patients who never started PEP or who were not compliant, was the high costs of the full PEP schedule. In Senegal, the costs of a full PEP schedule (excluding RIG) range from 40 to 60 euros, and according to the World Development Indicators, the average monthly disposable salary is around 160 euros [20]. In addition to the direct expenditure on PEP, costs from travel to distant PEP centres and lost income whilst seeking PEP were already reported in studies conducted in other rabies endemic countries [21–23]. Therefore, the true costs of PEP has been estimated to be twice as high as those reported in Africa [24] and should be considered in economic costs of rabies, as high costs might led to poor compliance with PEP regimens and increased risk of death [1]. The other main reason was the fact that the suspected animal was still alive at least 2 weeks after exposure, as reported by the patient and that the patient was informed by the Pasteur Institute of Dakar that PEP can be discontinued if, in the case of domestic dogs or cats, the animal remained healthy through a 2 weeks period of observation. With regards to this WHO recommendation, around 20% of the non-compliant patients of this study would have been considered as compliant. However, it was not possible to obtain a formal and well-documented assessment of the situation with a close follow-up of the health status of the animal after exposure because of absence of 10-day quarantine under the control of a veterinarian.

Thus, for clinicians, the decision to initiate a PEP is quite difficult, especially in a country where: i) there are large population of stray dogs, ii) no quarantine of suspected animals is implemented, and iii) laboratory testing for rabies confirmation in suspected animals is not regularly performed [25, 26]. This study estimates that around 5% of PEP was administered to patients whose risk for rabies would have been considered as low (such as exposure to healthy vaccinated dogs) based on the WHO recommendations [4, 27]. Thus, the implementation of the WHO recommendations should improve the number of patients completing PEP while reducing the unnecessary PEP for non-rabies exposures [27, 28]. However, the implementation of such recommendations in Senegal remains a challenge as the reliable data on the exposure (including epidemiological likelihood that the suspected animal was rabid, clinical features of the animal, its vaccination status or its availability for observation and laboratory testing) is currently rarely available for a full assessment of each individual case.

During this 1-year study, as observed in other Sub-Saharan African countries, the proportion of patients who received equine RIG among those patients who should have received it according to the WHO recommendations, was very low (less than 10%) mostly because of the costs [1] and the lack of supply [29, 30].

Based on the information reported by the patients or family (own perception), this study shows a good knowledge regarding the risks for rabies, how to prevent the disease, and where to receive rabies vaccination. This high level of self-reported awareness may be due to the availability of information from multiple sources, including recent government campaigns related to the national control and prevention program against rabies implemented in October 2010. However, a recent study showed measurable differences between the actual knowledge of rabies and the own perception of the patients, that raise an important issue, implying that the public may not be aware of their lack of knowledge [31]. Moreover, this high level of self-reported awareness could not be extrapolated to the general Senegalese population as the targeted population of this study was the selected patients who came to the Pasteur Institute of Dakar to seek a consultation for suspected rabies exposure.

Few patients reported adverse effects after PEP. Similar proportion of mild systemic adverse events (such as transient fever, headache) and pain at the site of injection, have been reported with rabies vaccines (5–15%) [32, 33]. In general, PEP is safe and well tolerated [27].

In order to increase the adherence to PEP, a free of charge prophylaxis might be implemented as it has already been done in other countries where rabies is endemic [8, 34]. Moreover, intradermal regimens, successfully introduced in some endemic countries [22, 34–36], should be considered as they can reduce the cost by about 70%, compared to intramuscular regimens [37, 38]. Moreover, these intradermal regimens have been found to be equally immunogenic and as effective as the standard intramuscular regimens [33, 37, 39–41].

Moreover, a careful risk assessment of each individual case following the WHO guidelines [27, 42, 43] would reduce the overuse or misuse of PEP, and therefore reduce expenditure. Therefore, trainings should be conducted to support Senegalese health workers in better clinical decision-making when administering PEP. We also recommend that bitten victims should receive a counselling with documented medical providers’ recommendations [28] and should be closely followed to ensure the compliance of PEP, by using either follow-up calls or short message service text messages [44].

## Conclusions

In Senegal, human rabies remains a major public health concern. The current prevention is accomplished by avoiding exposure to rabid animals or through PEP. Therefore, a national program for control and elimination of canine rabies was implemented in Senegal by the Ministry of Health since October 2010 with strengthening of collaboration between medical and veterinary sectors and investments in these two sectors. Regarding the PEP use and administration, this study shows that despite the public awareness about rabies dangers and prevention, only half of patients who received PEP at the Pasteur Institute of Dakar completed PEP. As PEP is a complex decision-making process for clinicians, especially when the suspected animal is not available for surveillance, recommendations such as free-of-charge prophylaxis or intradermal regimens should be considered to increase the adherence to PEP, as a complementary policy to prevent and control rabies in Senegal.

## Abbreviations

OR: Odds ratios
PEP: Post-Exposure Prophylaxis
RIG: Rabies immunoglobulin
WHO: World Health Organisation
